# Transvaginal Mini-Laparoscopic Splenectomy

**DOI:** 10.7759/cureus.336

**Published:** 2015-09-29

**Authors:** Mehmet Ali A Yagci, Cuneyt Kayaalp, Fatih Sumer

**Affiliations:** 1 Surgery, Inonu University

**Keywords:** laparoscopy, natural orifice surgery, splenectomy, transvaginal, natural orifice specimen extraction, natural orifice transluminal endoscopic surgery

## Abstract

We aimed to perform a more and more minimal invasive splenectomy by only through two 5 mm umbilical trocars and one vaginal trocar. A 43-year-old female (BMI 31 kg/m^2^, ASA II) with immune thrombocytopenic purpura was planned for splenectomy. She had a history of a previous cesarean section for three times. Two 5 mm trocars were inserted separately through the umbilicus. We did not use any single port device or similar modifications. A 15 mm trocar was inserted through the posterior fornix of the vagina under umbilical laparoscopic vision. The 5 mm umbilical ports were used for camera and retraction of the spleen. The transvaginal port was used for dissection and division of the spleen by a 10-mm LigaSure Atlas vessel sealing system. No clips or staples were used. As the spleen became completely free in the abdomen, it was removed through the vagina in a bag without fragmentation. The operating time was 200 minutes and the blood loss was minimal (< 20 ml). No drain or abdominal fascia suturing was used but closing the posterior fornix of the vagina. Her postoperative course was uneventful and she was discharged on day two without complication. She did not require any analgesics postoperatively. Platelet values increased to 408.000 mm^3^ in the follow-up. To the best of our knowledge, this report described the most minimal invasive splenectomy even. Additionally, it provided an unfragmented spleen extraction. The transvaginal approach seems to be a feasible way to perform natural orifice splenectomy.

## Introduction

Splenectomy is a common procedure for the management of spleen-related hematological disorders. Since laparoscopic splenectomy was first successfully performed by Delaitre in 1992, there has been a dramatic increase in the proportion of laparoscopic cases with acceptable outcomes. It became the “gold standard” for surgical treatment of some hematological disorders, such as immune thrombocytopenic purpura (ITP) [1]. Laparoscopic splenectomy is an effective and reliable technique, resulting with shorter hospital stays with fewer surgical complications and better esthetic results [2]. Mini-laparoscopic (< 5 mm trocars) surgery can also be used successfully for splenectomy; there will be less abdominal wall trauma that results in almost no risk of an incisional hernia, less postoperative pain, and a superior cosmetic outcome. There were a limited number of reported mini-laparoscopic splenectomies, and all had a 12 mm abdominal trocar for stapling or specimen extraction that made questionable the rationality of the mini-laparoscopy [3-4]. Natural orifice transluminal endoscopic surgery (NOTES), which is modified from laparoscopic surgery, reduces the number of ports and does not produce any visible surgical scar. A recent meta-analysis demonstrated that it has some advantages, such as less postoperative pain, low hernia risk, and faster recovery [5]. Despite those potential benefits, there were only two reported transvaginal splenectomies; both used flexible scopes, endostaplers for hilum transections, and extraumbilical trocars [1-2]. Herein, we demonstrated a laparoscopic splenectomy with rigid instruments without using staplers, clips, or sutures. We only placed two 5 mm umbilical trocars (mini-laparoscopy) and the spleen was removed through the vagina (natural orifice surgery).

## Case presentation

A 43-year-old female who initially presented with spontaneous nosebleeds was diagnosed with ITP and had been followed for three years with medical treatments. Her body mass index (BMI) was 31 kg/m2 and American Society of Anesthesiologists (ASA) score was II. She had previous cesarean sections X3. Physical examination revealed a Pfannenstiel incision. Her platelet counts were ranging between 1,000 and 26,000 mm^3^ at the beginning of the medical treatment, and she responded well to steroid therapy as rising to 203,000 mm^3^. During the ongoing medical treatment, steroid resistance developed and the patient was consulted for splenectomy. Her biochemical parameters were in normal ranges. Ultrasound indicated that the spleen was of normal in size.

The patient fully consented to the operation and also signed a detailed information consent form. Further, she was fully aware of the potential risks of the transvaginal approach, such as vaginal hemorrhage, infection, and dyspareunia, which were explained sufficiently to the patient.

**Figure 1:**
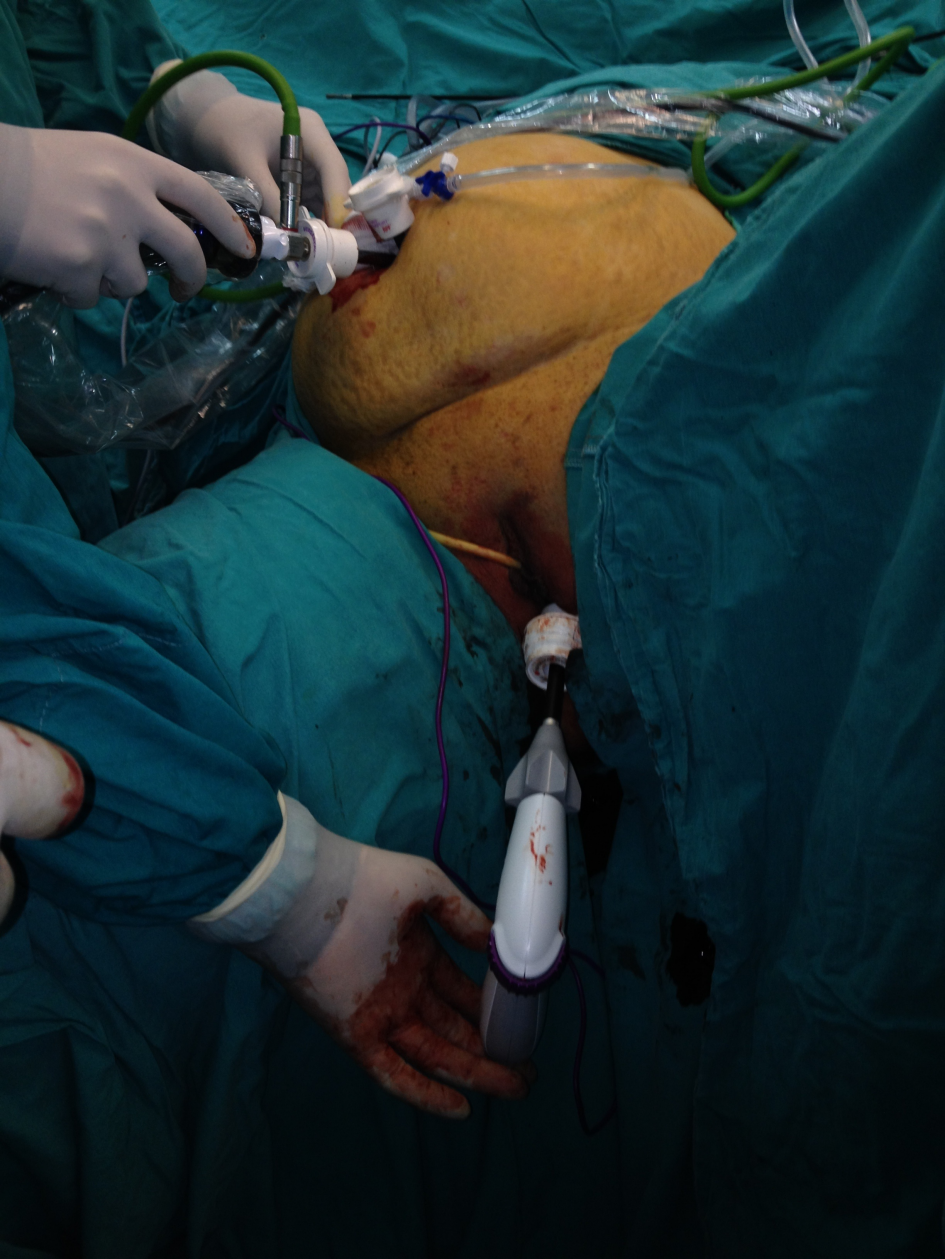
The position of the patient and trocars (2 x 5 mm umbilical and 12 mm vaginal).

Following induction of general anesthesia, all indicated monitoring lines (central venous pressure, arterial pressure, pulse oximetry, electrocardiogram, and blood pressure cuff) were placed and secured. An upper body warming device was laid across the patient’s chest to help maintain normothermia. A urinary bladder catheter and a nasogastric tube were also inserted. The patient was placed on the operating table in 30° right lateral decubitus position with the legs abducted and slightly flexed at the knees. The abdomen, pelvis, and vaginal canal were disinfected with povidone-iodine. The camera assistant stood on the right side of the patient. The surgeon stood between the patient’s legs. Pneumoperitoneum was created by a Veress needle from the left subcostal area. A transverse 5 mm skin incision was made into the umbilicus, and a 5 mm trocar was inserted into the abdomen. The insufflator was set to a pressure of 14 mm Hg. A laparoscopic exploration showed no abdominal adhesions. One more 5 mm port was inserted through the umbilicus through a separate skin and fascia incision. The uterus was grasped for good visualization of the pelvis. The patient was then placed in a steep Trendelenburg position to allow adequate exposure for the transvaginal access. A 15 mm trocar was inserted through the posterior fornix of the vagina under laparoscopic vision. The umbilical ports were used for laparoscopic vision, retraction, and elevation of the spleen from caudal to cranial. All dissections of the spleen and sealing of the hilar vessels and short gastric vessels were done with a 10 mm LigaSure Atlas XL vessel sealing system (Valleylab, Boulder, CO, USA) through the transvaginal port (Figure 1).

**Figure 2:**
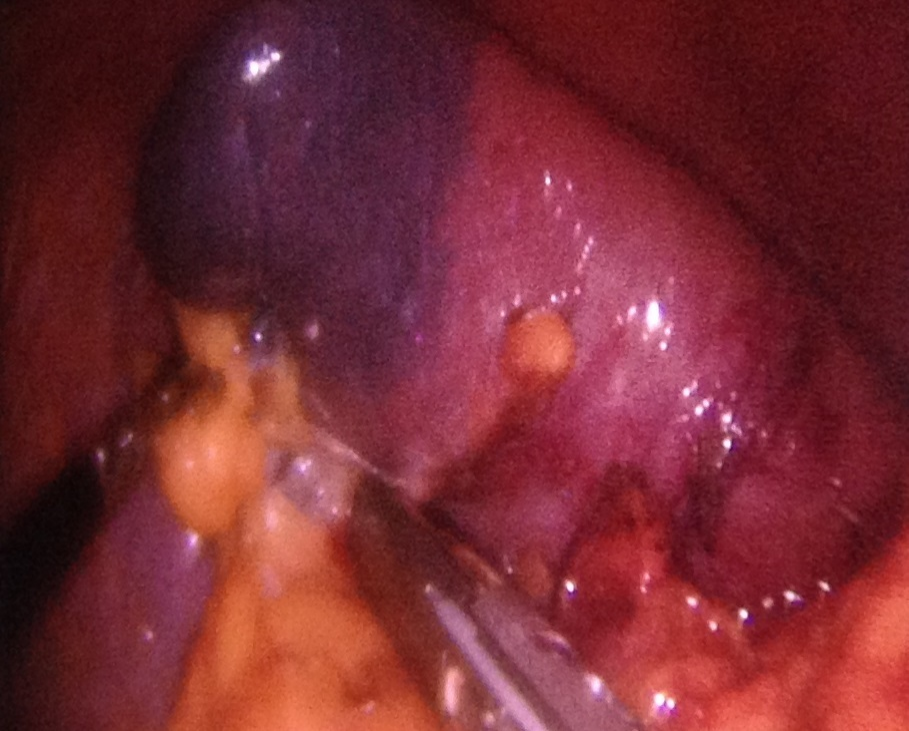
Retraction of the spleen by umbilical trocar and division by transvaginally placed Ligasure vessel sealing system.

No laparoscopic clips, sutures, or monopolar–bipolar diathermy were used. After looking for accessory splenic tissues, dissection and sealing of the splenic vessels was performed consequently from the lower pole to the upper pole and from the lateral to medial (Figure 2).

**Figure 3:**
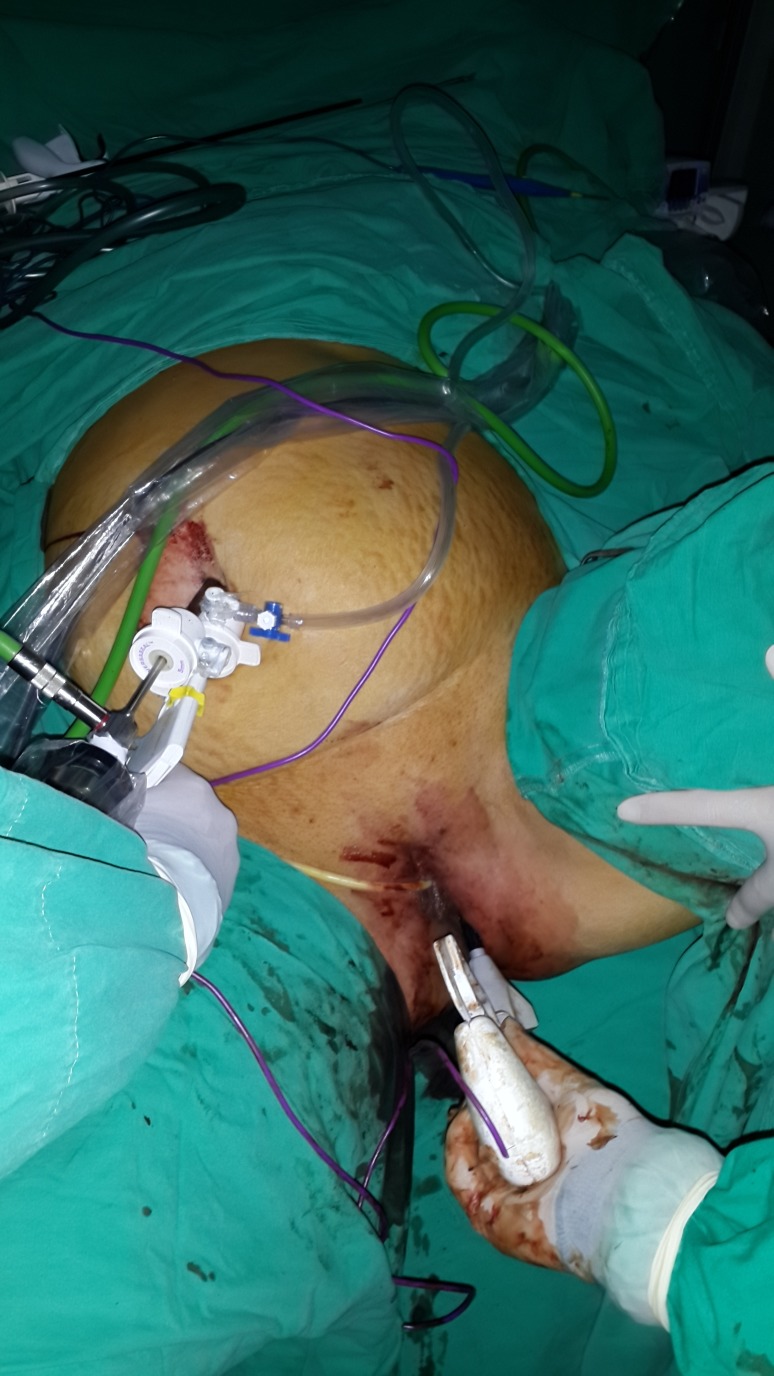
Transvaginal use of Ligasure XL after removal of the vaginal trocar.

When approaching the hilum of the spleen, the length of the Ligasure was not sufficient. At this point, the 15 mm vaginal trocar was removed and a Ligasure device introduced through the vaginal port without the trocar (Figure 3).

**Figure 4:**
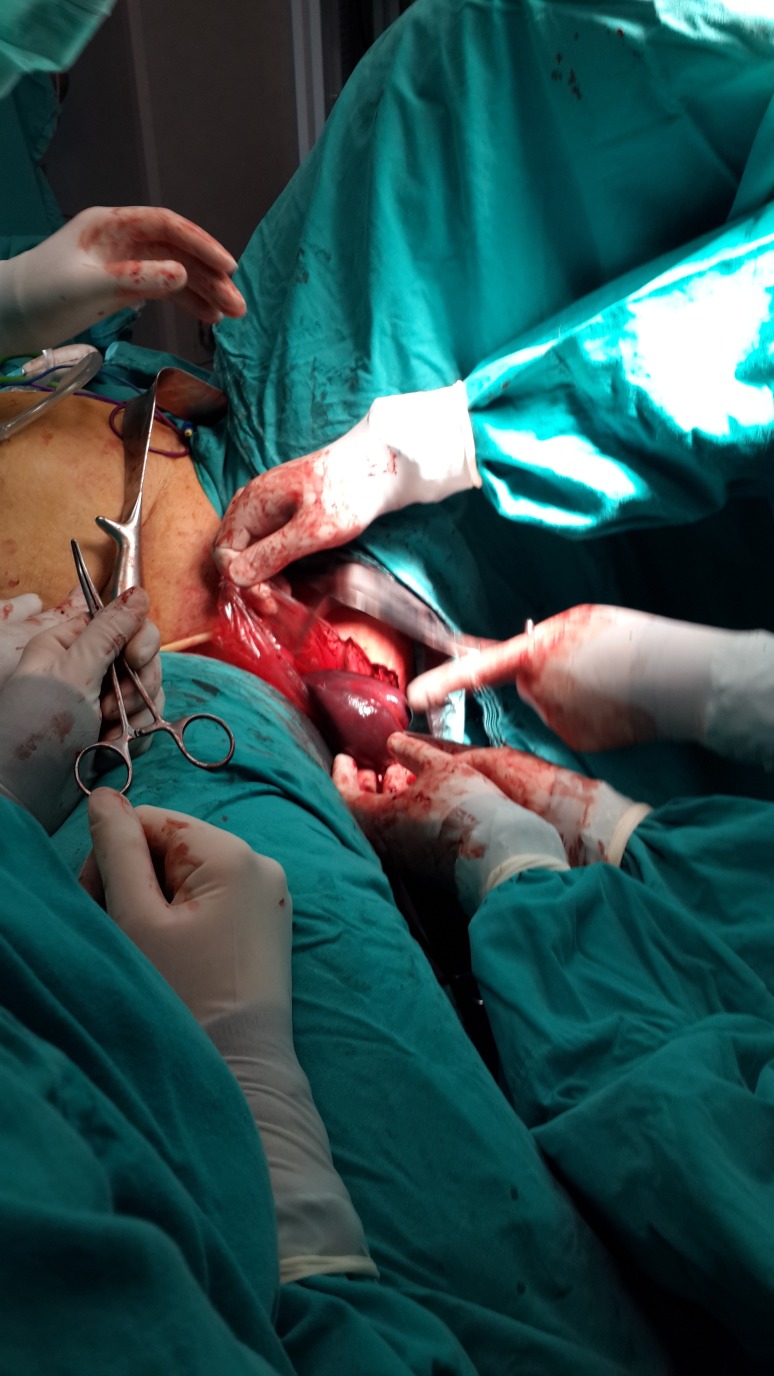
Transvaginal extraction of the spleen.

**Figure 5:**
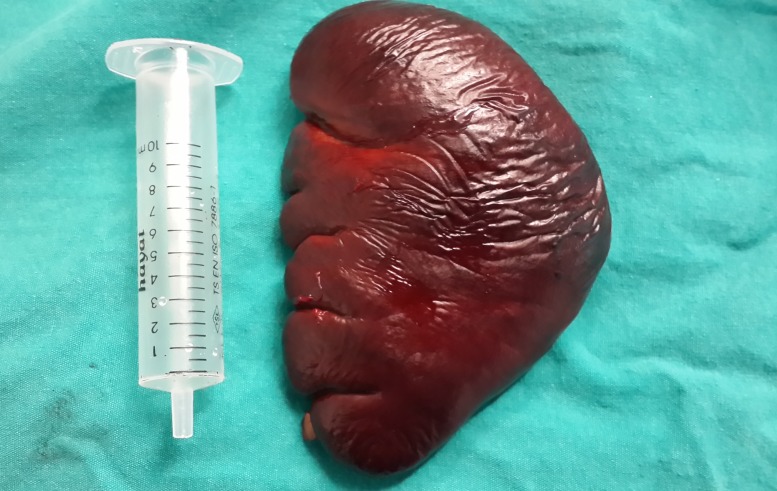
Unfragmented spleen removed by laparoscopic technique.

In this way, the Ligasure could reach to the left diaphragm. Hilar vessels and short gastric vessels were divided close to the splenic capsule, and finally, splenophrenic ligaments were released. The techniques of opening the gastrosplenic ligaments, early ligation of the splenic artery, or dissection of the pancreatic tail were not used. The spleen was placed in a large specimen bag (Endocatch, Tyco, US) and removed from the abdomen through the 15 mm vaginal port without fragmentation after the dilatation of the posterior fornix with fingers (Figures 4-5).

**Figure 6:**
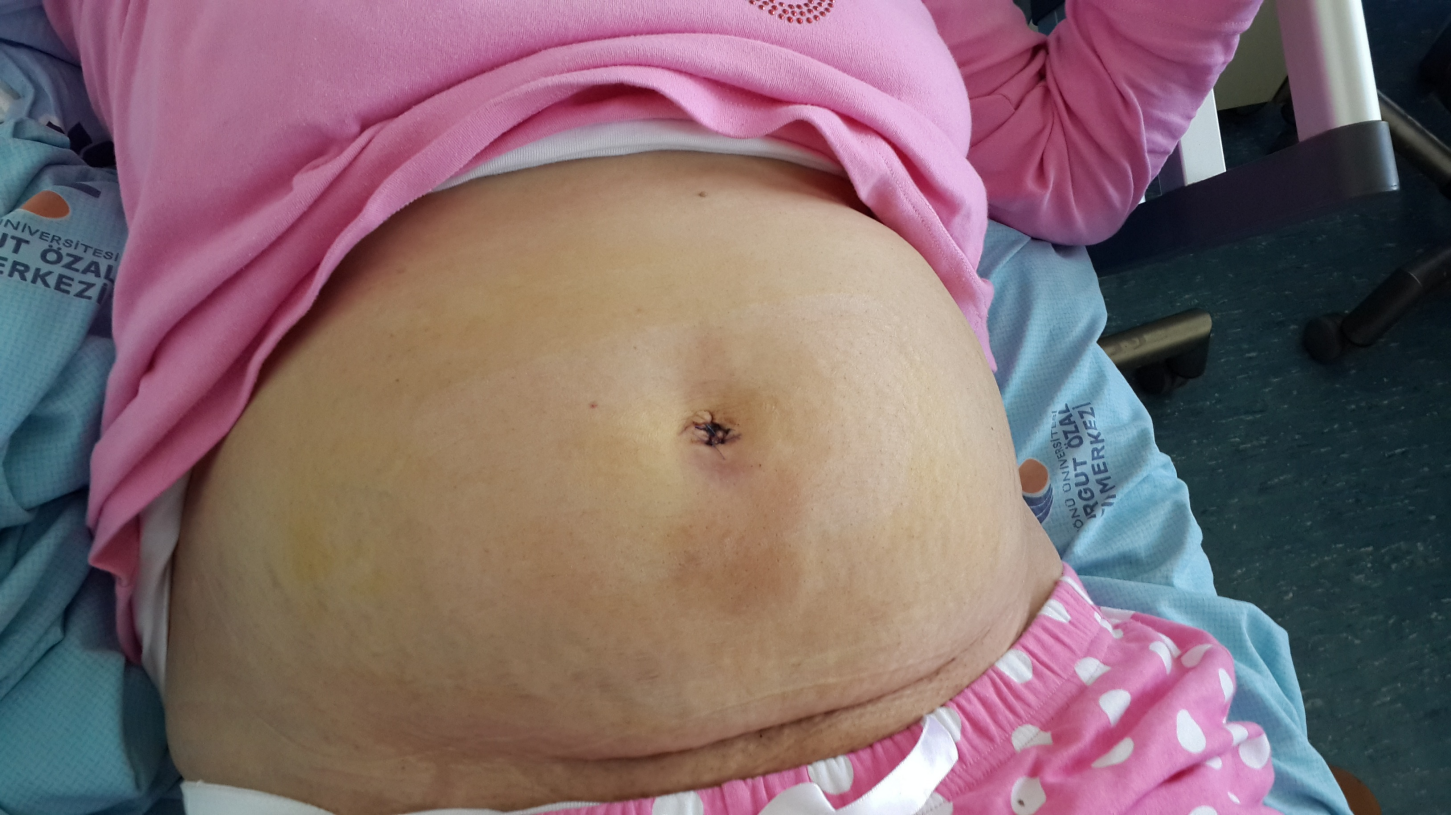
Postpoperative view of the abdomen without visible abdominal scars.

The vagina was irrigated with a povidone–iodine solution. The colpotomy was then closed with a running absorbable suture through the vagina after a strict hemostasis. A povidone–iodine-soaked vaginal pack was placed into the vagina for 12 hours. Neither drain nor fascial suturing was used. The umbilical incisions were closed with non-absorbable sutures (Figure 6).

The operating time was 3 hours 20 minutes. The blood loss was minimal (< 20 ml). The nasogastric tube was removed the night of surgery. The patient was allowed to drink fluids and a soft oral diet on the first postoperative day. The postoperative course was uneventful. The patient was discharged on the second postoperative day without wound infection, fever, pain, urinary difficulty, or other specific complication. No analgesics were administrated during the postoperative period. At the ambulatory follow-up observation performed four weeks after discharge, the abdominal and vaginal wound had healed well, and the patient had no specific complaints. Her platelet count was 408.000 mm^3^. The histopathological examination showed an ordinary spleen with 140 grams and 11 × 9 × 3 cm in size.

## Discussion

Here, we described a laparoscopic transvaginal splenectomy in a patient with ITP. Unlike the previously reported two transvaginal splenectomy cases, we did not use flexible endoscopes or endoscopic staplers [1-2]. The disadvantage of a current flexible endoscope is being unstable along their axis, which makes positioning maneuvers difficult in the surgical field. Targarona, et al. solved this problem by using an extra 3 mm port to hang the tip of the scope in front of the operative area [2]. In our initial case, the transvaginal splenectomy with rigid instruments was technically feasible.

One important challenge of transvaginal surgery is the safety of vaginal access. Pure transvaginal approaches use blind access through the vagina, and it has the possibility of adjacent organ injuries. Wood, et al. reported a rectal injury due to blind vaginal access during a pure transvaginal ventral hernia repair [6]. We believe that trocar insertion through the vagina under laparoscopic guidance is safer (Table 1).

**Table 1:** Details of the previously published two transvaginal splenectomies and our case.

	Targarona (2009)	Trenard (2011)	Present Case (2014)
No. of patients	1	1	1
Country	Spain	Venezuela	Turkey
Age (years)	65	30	43
Body mass index (kg/m^2^)	30	22	31
Previous surgery	No	Yes	Yes
Indication	Cystic tumor	ITP	ITP
Spleen size	12 cm	15 x 10 cm	11 x 9 x 3 cm
Surgical technique	Hybrid	Hybrid	Hybrid
Patient position	Lateral	Semilateral	Semilateral
Number of abdominal trocars	4	2 (5-12 mm umbilical)	2 (2 x 5 mm umbilical)
Abdominal trocars except umbilicus	Yes (3 x 5 mm)	No	No
Any single port access equipment	No	No	No
Scope type	Flexible	Flexible	Rigid
Perisplenic dissection port	Abdominal	Abdominal	Vaginal
Perisplenic dissection equipment	Harmonic scalpel	Harmonic scalpel	Ligasure
Splenic hilum transection port	Vaginal	Abdominal	Vaginal
Splenic hilum transection equipment	Endo-Stapler	Endo-Stapler	Ligasure
Fragmentation of spleen	No	Yes	No
Weight of spleen (gram)	178	168	140
Operating time (minutes)	180	150	200
Blood loss (ml)	< 50	100	< 20
Additional port requirement	No	No	No
Conversion	No	No	No
Complication	No	No	No
Hospital stay (hours)	48	48	48

We used two 5 mm umbilical ports for laparoscopic vision and retraction of the spleen. The main advantages of this technique were that it reduces the port numbers, avoids penetration of the abdominal wall with 10-15 mm trocars, and eliminates visible scars, hernia risk, and fascial suturing that increases the postoperative pain. Our patient did not require any postoperative analgesics and there was no incision-related complication. Insertion of the endobag and extraction of the spleen without any fragmentation through the Douglas pouch did not cause any difficulty. Additionally, extraction of the spleen without fragmentation is clearly beneficial in some splenic pathologies [7].

Despite those advantages, there are still some obstacles with this technique. Handles of two umbilical trocars crossed to each other and resulted to their inability to work at the same direction. The distance between the spleen and the vagina and the inadequate length of the instruments was another difficulty. Previously, we demonstrated the safety of vessel sealing system (Ligasure) for all vascular controls in laparoscopic splenectomy [8]. However, the length of the device was not sufficient when approaching the hilum of the spleen. We gained length by removing the vaginal trocar and then the whole shaft of the Ligasure could then be used. Direct insertion of the Ligasure through the vagina did not result in failure of pneumoperitoneum, but transvaginal Ligasure insertion without trocar caused the inability to change the transvaginal equipment with another. We think that the limitations of this approach are clearly technology-dependent and could be more comfortable by the development of new endosurgical equipment.

## Conclusions

To the best of our knowledge, this report describes the most minimal invasive splenectomy ever. Additionally, it provided an unfragmented spleen extraction. As a conclusion, the transvaginal splenectomy seems to be a feasible and effective approach to performing NOTES. However, before its wide acceptance, some developments in technology are still necessary.
